# A reflection on frustrated Lewis pairs 20 years on: the gift that keeps on giving

**DOI:** 10.1039/d5sc90195b

**Published:** 2025-09-24

**Authors:** Rebecca L. Melen, Douglas W. Stephan

**Affiliations:** a Cardiff Catalysis Institute, School of Chemistry, Cardiff University, Translational Research Hub Maindy Road, Cathays Cardiff Cymru/Wales CF24 4HQ UK MelenR@cardiff.ac.uk; b Department of Chemistry, University of Toronto 80 St. George St. Toronto Ontario M5S3H6 Canada douglas.stephan@utoronto.ca

## Abstract

This commentary reflects on the remarkably broad impact the concept of “frustrated Lewis pairs” (FLPs) has had over the past 20 years. Since its initial articulation, this concept has found applications across the periodic table and the discipline, leading to new avenues for synthesis and catalysis, building on the 2011 publication (R. C. Neu, E. Otten, A. Lough and D. W. Stephan, *Chem. Sci.*, 2011, **2**, 170, https://doi.org/10.1039/C0SC00398K).

## Abstract 2

In celebration of our 15th anniversary and some of our most popular articles, Douglas W. Stephan and Rebecca L. Melen reflect on the area of frustrated Lewis pairs, building on the paper published in *Chemical Science* (D. W. Stephan *et al.*, *Chem. Sci.*, 2011, https://doi.org/10.1039/C0SC00398K).

## Graphical abstract

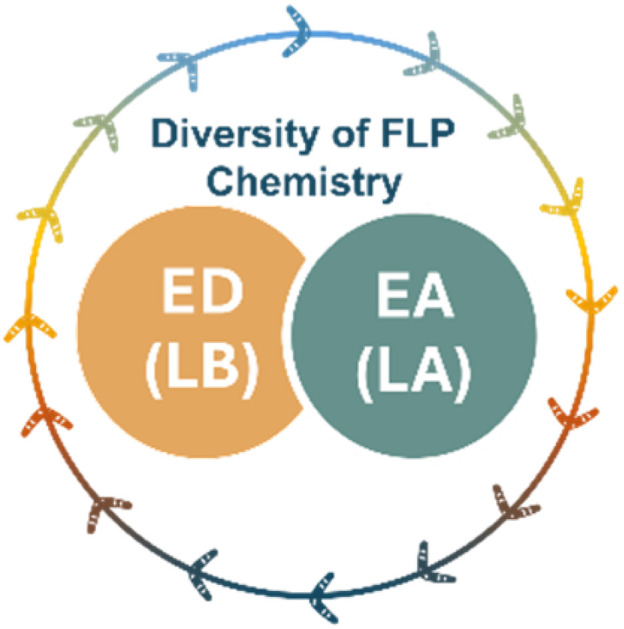

In some cases, an insight into reactivity can take scientists down unexpected roads to new perspectives, new approaches, ultimately delivering a broader approach to chemical applications and utility. Over the last 20 years, this has certainly been the case for the concept of “frustrated Lewis pairs” (FLPs) which, as illustrated in Fig. 1 has impacted many areas of modern chemistry. This concept emerged almost twenty years ago, based on the observation that phosphino-borane Mes_2_PC_6_F_4_B(C_6_F_5_)_2_ acted as the first metal-free species capable of reversible activation of H_2_.^1^ Shortly thereafter it was recognized that this reactivity was not limited to this phosphino-borane, but could be achieved with combinations of donors and acceptors. While it was initially thought to be limited to cases where dative interactions were sterically inhibited, in fact this reactivity has proven to be much more general as only equilibrium access to the free donor and acceptor is required.^2^ Moreover, such systems proved capable of reaction with other small molecules such as olefins and alkynes, prompting the descriptor “frustrated Lewis pairs” (FLPs).^3^ These early findings have provided a fertile basis upon which a remarkably broad range of chemistry has continued to grow.

**Fig. 1:**
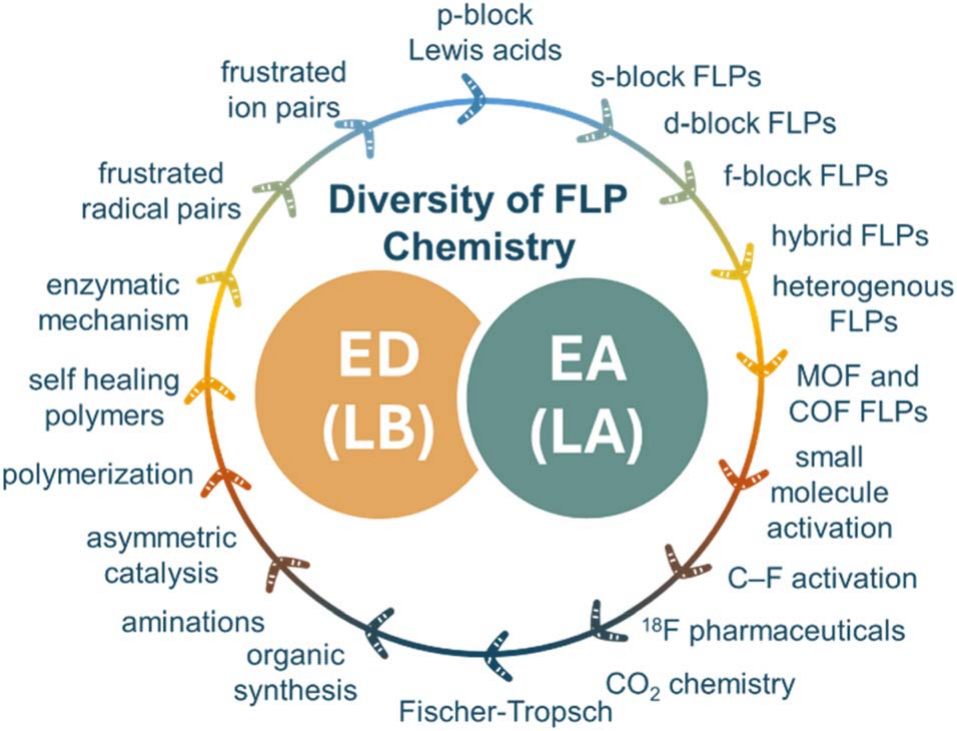
The diversity of chemistry emerging from FLPs. ED = electron donor; EA = electron acceptor; LB = Lewis base; LA = Lewis acid.

The first targets focused on the unique reactivity of FLPs with H_2_, leading to the emergence of metal-free hydrogenation catalysis,^4^ a notion that contravened a century of chemical dogma. While early studies focused on the substrate scope and mechanistic understanding, it quickly became obvious that this avenue to reduction could be applied to a broad range of organic substrates. Moreover, efforts to adapt FLPs to asymmetric reductions rapidly evolved, ultimately achieving high enantioselectivities in many cases.^5^ Nonetheless, like transition metal catalysts, efforts continue to broaden the range of substrates, improve catalyst efficiency and facilitate accessibility of novel and/or chiral catalysts.^6^

Another avenue that emerged from the initial studies was the reactivity of FLPs with small molecules in general. This prompted studies of a variety of main group FLPs with CO_2_, SO_2_, CO, RNSO, NO and N_2_O among other substrates (https://doi.org/10.1039/C0SC00398K).^7^ While these findings provided access to unique linkages and main group heterocycles, they also generated systems that have warranted further study. For example, while the capture of CO_2_ was interesting, several studies since have targeted stoichiometric and catalytic reductions. These have led to both new insights into the reaction mechanisms, as well as strategies to generate CO, methanol or methane.^7*b*,8^ In another example, the FLP capture of NO provided a stable radical. This species proved to be useful as a catalyst for radical polymerizations.^9^ One other example emerged from the demonstration that FLPs activated C–F bonds. This has been exploited to not only selectively fluorinate organic substrates but also to produce ^18^F radiopharmaceuticals.^10^

Recognizing that the chemistry of FLPs was not limited to traditional Lewis acids or bases, broadened the notion of FLP reactivity dramatically. One approach was to probe non-conventional main group Lewis acids.^11^ For example, although the Lewis acidity of group 15 species was known, exploration in the context of FLP chemistry led to the advent of highly electron deficient P(v) fluoro-phosphonium cations which act as strong σ*-Lewis acid acceptors.^12^ These species were exploited for C–F bond activations and functionalization, and as catalysts for FLP hydrosilylation and hydrogenations.^13^ Similarly, the Lewis acidity of P(iii) species in FLP reactions has also been uncovered. For example, the reaction of triphosphabenzene with H_2_ was shown to proceed *via* an FLP mechanism, involving a phosphorus and carbon atom in the 1,4-positions.^14^ Another more recent example used donor-stabilized P(iii) phosphenium cations as a source of the Lewis acid and base in FLP additions to alkynes. This provided a facile route to dissymmetric bidentate phosphine ligands, a class of ligands that are otherwise challenging to access.^15^

In further related expansion of the range of FLP chemistry, s-block element compounds have shown FLP behaviour. The cooperative action of the Lewis acidity of s-block cations with the basicity of the anion was demonstrated with both group 1 (ref. 16) and group 2 (ref. 17) derivatives, and have been shown to reversibly activate H_2_ and act as hydrogenation catalysts. Such species also react with small molecules, as reaction with syngas (CO/H_2_), led to concurrent homologation and reduction of CO. This demonstrated a transition metal-free approach to the fundamental steps of Fischer–Tropsch chemistry.^18^

The notion of FLPs has even infiltrated transition metal chemistry in several fashions.^19^ Bercaw was the first to exploit a P/B ligand to assist in the reduction of a Re-carbonyl complex in the presence of a strong base.^20^ This concept was subsequently shown to be pertinent to the enzymatic activation of H_2_ by [Fe] hydrogenase. Model studies confirmed that this activation is mediated *via* an FLP-type mechanism between the Lewis acidic Fe-centre and a pendant nitrogen donor.^21^ Another strategy expanded FLP chemistry by exploitation of the Lewis acidity or basicity of metal-based complexes themselves.^22^ Thus a range of electron rich, Lewis basic or electron deficient Lewis acidic metal complexes in combination with a main group counterpart, generated FLPs which activate a range of small molecules and provide catalytic systems. While this was initially confined to the d-block metals, this has recently been extended to the f-block. For example, a trivalent uranium complex and a silylene were shown to activate H_2_*via* an FLP mechanism, ultimately providing an avenue to the catalytic hydrogenation of silylenes.^23^ In a further extension of the concept of FLPs, combinations of Lewis acidic and basic metal complexes have also been shown to exhibit FLP reactivity.^24^

Targeting heterogeneous catalysis, a number of researchers have incorporated FLPs into MOFs or COFs, affording robust and selective catalysts for various transformations.^25^ Moreover the concept of FLPs has provided a new perspective on heterogeneous catalysts,^26^ as vacancies on the surface provide proximal, unquenched electron deficient and rich sites. This concept has been used to both understand, design and improve the efficiency of catalytic systems.

Another powerful aspect of FLP chemistry that has emerged is the utility in organic synthesis. Apart from reduction, the ability of FLPs to activate unsaturated organic substrates in facile and unique ways, has led to a myriad of new synthetic protocols, enriching the chemists' toolbox. For example, in the case of alkynes, reactions with a large range of FLPs has provided access to wide variety of heteroatom derived acyclic and heterocyclic products incorporating new C–P, C–N, C–O, C–S and C–C bonds among others.^27^ In addition, further FLP chemistry of alkynes has also enabled metal-free routes to hydroamination, hydrophosphination, hydroarylation and iodoperfluoroalkylation, in addition to avenues to alkyne dimerization, cyclization and dehydrocoupling catalysis.^28^ FLPs are also advancing frontiers in the persistent challenges of C–H bonds activation and asymmetric catalysis. For example, while the use of FLPs to effect C–H functionalization began with borylations,^29^ this has been widely extended (*vide infra*). Similarly, apart from the aforementioned use of chiral FLPs in hydrogenation, chiral FLPs have also been applied to effect the stereoselectivity of cycloadditions and ring opening reactions.^27^ Many of these systems rival traditional catalysts without metal contamination, a feature critical for pharmaceutical applications.

Another rich avenue for synthetic chemistry emerged from the recognition^30,31^ that some FLPs do not react in a two-electron process but rather, *via* a single electron transfer from the base to the acid, generating a “frustrated radical pair” (FRP).^32^ This finding has been applied to develop FRP-based synthetic procedures.^32*b*^ For example, the FRP derived from Mes_3_P/B(C_6_F_5_)_3_ was shown to activate the C–O bond of benzhydryl esters allowing addition to olefins, resulting in a new strategy for C–C bond formation.^33^ Adopting a related approach using the FRP derived from the mixture of *N*-methyl-*N*-((trimethylsilyl)methyl)aniline and B(C_6_F_5_)_3_, allowed the desilylative α-aminomethylation of Michael acceptors.^34^ Other applications have included FRP routes to the hydroboration of alkenes, intramolecular aminations, and the deoxygenation and azido-oxygenation of alkenes. Most remarkably, this strategy has been applied to one of the holy-grails of organic chemistry, selective sp^3^-C–H activations.^35^ In this case, FRPs derived from disilazide donors and an *N*-oxoammonium acceptor were used to selectively activate C–H bonds furnishing aminoxylated products. The nature of the donor was shown to control regioselectivity to allow selective reactivity at tertiary, secondary or primary C–H bonds.^35^ This opens up potential for late-stage functionalization of complex molecules, a key goal in drug discovery and agrochemical synthesis.

More recently, a distinct approach using frustrated ion pairs has emerged. Derived from the combination of phosphonium salts and a lithium amide, electron transfer in the presence of an alkylhalide effects the formation of new C–C bonds.^36^ As the initial phosphonium salts are generated from alkyl halides, the net result is the coupling of unactivated electrophiles and is a process that tolerates functional groups that are challenging for transition-metal-catalysts.^36^

A variety of other avenues of application of FLPs have also emerged in the general area of polymer chemistry. Here their high reactivity, coupled with the ability to operate under mild and tunable conditions, makes them attractive candidates for producing novel polymers with fine-tuned architectures, offering a pathway to the metal-free synthesis of functional materials. For example, FLPs have been used as catalysts for polymerization catalysis^37^ providing access to ultrahigh molecular weight poly-methyl-methacrylate.^38^ Alternatively, an innovative new class of self-healing polymers have been derived from the combination of two polymers containing pendant phosphines and boranes, respectively.^39^ Related polymers have also found application in catalysis^40^ and for the generation of polymeric FRPs.^41^

One can view the discovery of FLPs as a corollary to the century old Lewis acid-base theory. It is pleasing in its simplicity and thus readily allows undergraduates to extend Lewis' concepts to reactivity in a new way. At the same time, detailed studies by researchers have uncovered the subtleties and inferences that have led to innovations, developments and applications of the concept across the periodic table. While the last 20 years have firmly established the term “FLP” in the lexicon of chemists, it has also provided access to a remarkable diversity of new protocols in synthesis and catalytic chemistry across the discipline. FLPs remain a source of ongoing innovation, and this firmly established paradigm will undoubtedly inspire future advances.

## Author contributions

DWS drafted the manuscript and RLM edited it.

## Conflicts of interest

There are no conflicts to declare.

## Data Availability

No data was generated as part of this manuscript.
